# Accuracy of Curable Sexually Transmitted Infections and Genital Mycoplasmas Screening by Multiplex Real-Time PCR Using a Self-Collected Veil among Adult Women in Sub-Saharan Africa

**DOI:** 10.1155/2019/8639510

**Published:** 2019-07-15

**Authors:** Zita Aleyo Nodjikouambaye, Fabrice Compain, Damtheou Sadjoli, Ralph-Sydney Mboumba Bouassa, Hélène Péré, David Veyer, Leman Robin, Chatté Adawaye, Serge Tonen-Wolyec, Ali Mahamat Moussa, Donato Koyalta, Laurent Belec

**Affiliations:** ^1^Service de Gynécologie-Obstétrique, Hôpital de la Mère et de l'Enfant, N'Djamena, Chad; ^2^Ecole Doctorale Régionale en Infectiologie Tropicale de Franceville, Franceville, Gabon; ^3^Laboratoire de Microbiologie, Hôpital Européen Georges Pompidou, Paris, France; ^4^INSERM UMRS 1138, UPMC Université (Paris VI), Université Paris Descartes (Paris V) and Université Paris Diderot (Paris VII), Centre de Recherche Biomédicale des Cordeliers, Paris, France; ^5^Faculté de Médecine Paris Descartes, Université Paris Descartes (Paris V), Sorbonne Paris Cité, Paris, France; ^6^Cabinet Médical de Gynécologie-Obstétrique “La Renaissance Plus”, N'Djamena, Chad; ^7^Institut National Supérieur des Sciences et Techniques d'Abéché, Abéché, Chad; ^8^Faculté de Médecine, Université de Bunia, Bunia, Democratic Republic of the Congo; ^9^Faculté de Médecine et de Pharmacie, Université de Kisangani, Kisangani, Democratic Republic of the Congo; ^10^Faculté de Médecine, N'Djamena, Chad; ^11^Service de Gastro-Entérologie, Hôpital Général de Référence Nationale, N'Djamena, Chad; ^12^UNAIDS, N'Djamena, Chad

## Abstract

**Background:**

Sexually transmitted infections (STIs) are highly prevalent in sub-Saharan Africa. Genital self-sampling may facilitate the screening of STIs in hard-to-reach remote populations far from large health care centers and may increase screening rates. The cross-sectional* GYNAUTO-STI* study was carried out to assess the performance of a novel genital veil (V-Veil-Up Gyn Collection Device, V-Veil-Up Pharma, Ltd., Nicosia, Cyprus) as a genital self-sampling device to collect genital secretions to diagnose STIs by molecular biology as compared to reference clinician-collected genital specimens, in adult African women.

**Methods:**

Adult women living in N'Djamena, the capital city of Chad, were recruited from the community and referred to the clinic for women's sexual health “*La Renaissance Plus*”. A clinician obtained an endocervical specimen using flocked swab. Genital secretions were also obtained by self-collection using veil. Both clinician- and self-collected specimens were tested for common curable STIs (including* Chlamydia trachomatis*,* Neisseria gonorrhoeae*,* Mycoplasma genitalium*, and* Trichomonas vaginalis*) and genital* Mycoplasma *spp. by multiplex real-time PCR (Allplex™ STI Essential Assay, Seegene, Seoul, South Korea). Test positivities for both collection methods were compared by assessing methods agreement, sensitivity, and specificity.

**Results:**

A total of 251 women (mean age, 35.1 years) were prospectively enrolled. Only seven (2.8%) women were found to be infected with at least one common STIs [*C. trachomatis*: 3 (1.2%),* N. gonorrhoeae*: 1 (0.4%),* M. genitalium*: 4 (1.6%) and* T. vaginalis*: 1 (0.4%)], while the prevalence of genital mycoplasmas was much higher (54.2%) with a predominance of* Ureaplasma parvum* (42.6%). Self-collection by veil was non-inferior to clinician-based collection for genital microorganisms DNA molecular testing, with “almost perfect” agreement between both methods, high sensitivity (97.0%; 95%CI: 92.5-99.2%), and specificity (88.0%; 95%CI: 80.7-93.3%). Remarkably, the mean total number of genital microorganisms detected per woman was 1.14-fold higher in self-collected specimens compared to that in clinician-collected specimens.

**Conclusions:**

Veil-based self-collection of female genital secretions constitutes a convenient tool to collect in gentle way cervicovaginal secretions for accurate molecular detection of genital bacteria. Such sampling procedure could be easily implemented in STIs clinics in sub-Saharan Africa.

## 1. Introduction

Worldwide sexually transmitted infections (STIs) stand as a major global health concern, and more than a million of STIs are acquired per day [1]. Common bacterial and parasitic STIs conveyed by* Chlamydia trachomatis*,* Neisseria gonorrhoeae*,* Mycoplasma genitalium*, and* Trichomonas vaginalis* could be easily treated if properly detected. The World Health Organization (WHO) reports global numbers worldwide of new cases for* C. trachomatis*,* N. gonorrhoeae*, and* T. vaginalis* infections of 78, 131, and 142 million every year, respectively [2]. According to WHO estimations, sub-Saharan Africa supports 40% of the global burden of STIs and covers 44% of the need for services and 30% of global control cost related to STIs [2, 3]. However, the prevalence of STIs in sub-Saharan Africa is highly heterogeneous, and a systematic review performed on behalf of the WHO reported slightly low rates in 15–49-year-old African women from the general population (*N. gonorrhoeae*, 1.7%;* C. trachomatis*, 3.7%;* T. vaginalis*, 11.5%) [4].

Routine screening is an important component of STIs prevention and control. However, it has been recognized that an effective response to the global STIs epidemic necessitates alternative strategies beyond the traditional approach of clinic-based sampling and conventional culture methods for screening [5, 6]. Microbiological screening must be incorporated into STIs control strategies. However, lack of access to appropriate services (especially in rural and remote areas), reluctance of at-risk populations to attend for treatment, fear of invasive genital examinations, and lower sensitivities of conventional diagnostic assays reduce the effectiveness of clinic-based screening programs that capture only a small proportion of people with STIs [5, 6]. The advent of new technologies such as nucleic acid amplification tests (NAATs) has provided opportunities for expansion of screening for STIs in general population. Furthermore, self-sampling using non-invasive or minimally invasive sampling techniques such as urine samples, cervical-brush, vaginal swabs, and tampons may be easily carried out individually by women at home without special medical qualification and special assistance [5] and allows women to preserve their intimacy [7]. Specimens for NAATs can be stored at room temperature while being transported to the laboratory. Self-sampling may facilitate the screening of STIs in hard-to-reach remote populations far from large health care centers. Because of savings on the cost of clinical overheads, home-sampling STIs testing services may be more cost-effective compared with traditional services. Finally, over the past two decades, several studies in Western countries have reported on high acceptability of home sampling with equivalent or superior number of screening diagnosis completed for chlamydia and gonorrhea as compared to conventional sampling and detection methods [5, 7–14].

Limited data currently exist on the performance of genital self-sampling for STIs detection by NAATs in general female population living in resource-constrained countries [15, 16]. The main objective of the present study, so-called* GYNAUTO-STI *operational research project, was to assess the performance of a novel genital veil (V-Veil-Up Gyn Collection Device, V-Veil-Up Pharma Ltd., Nicosia, Cyprus) as a genital self-sampling device to collect genital secretions to diagnose STIs by multiplex real-time PCR as compared to reference clinician-collected genital specimens, in adult women living in N'Djamena, the capital city of Chad. Chad is a central African country of 15 million inhabitants, mostly young women [17, 18], where the epidemiology of common bacterial STIs remains undocumented. Thus, the study was also conceived to provide epidemiological data on STIs shedding in asymptomatic adult women living in Chad.

## 2. Methods

### 2.1. Study Design

The cross-sectional* GYNAUTO-STI *study compared the bacteria DNA diagnostic accuracy of a clinician-collected endocervical sample with swab and a cervicovaginal self-collection method with veil device in adult women living in N'Djamena, Chad, recruited from the community. The 2015 Standards for Reporting of Diagnostic Accuracy (STARD) guidelines were used for reporting the study [19, 20].

### 2.2. Enrolment and Selection Criteria

The capital city N'Djamena comprises 10 districts, which include a variable number of neighborhoods. Twenty-three sites in neighborhoods of 5 districts randomly selected out of 10 were further chosen for study inclusion [1^st^ district: Farcha, Amsinéné, Karkandjeri; 3^rd^ district: Gardolé, Ardep Djoumal, Kabalaye, Sabangali; 6^th^ district: Moursal, Paris-Congo; 7^th^ district: Ambata, Amtoukouin, Atrone, Boutalbagara, Chagoua, Dembé, Gassi, Habena, Kilwiti; 9^th^ district: Digangali, Gardolé, Ngueli, Toukra, Walia]. In each inclusion site, peer educators contacted adult women in community churches and mosques or women association networks during a one-month period and proposed them to be included in the* GYNAUTO-STI *study after an oral explanation and collective awareness sessions on the objectives of the survey, mainly focused on sensitization on prevention strategies against STIs and cervical cancer. After oral consent, the selected women were invited, with paid transportation, to come to the clinic “*La Renaissance Plus*”, N'Djamena, which is one of the main settings for women's sexual health in Chad, and to participate in the study. Childbearing-aged and older women living in N'Djamena regularly attend the clinic “*La Renaissance Plus”* for gynecological examinations and for obstetrical services.

The inclusion criteria were being a volunteer, having given signed informed consent, being more than 18 years, being sexually active, having no genital troubles at physical examination, not menstruating, having no sexual intercourse for at least 48 hours, and having completed the questionnaire. Exclusion criteria included age less than 18 years, having genital troubles, having menstruations, having recent sexual intercourse less than 48 hours, and not willing to participate to the study or to answer the face-to-face questionnaire to collect data. Note that the menstrual cycle phases were not taken into account.

### 2.3. Clinical Visit Procedures and Genital Samples Collection

**Figure 1:**
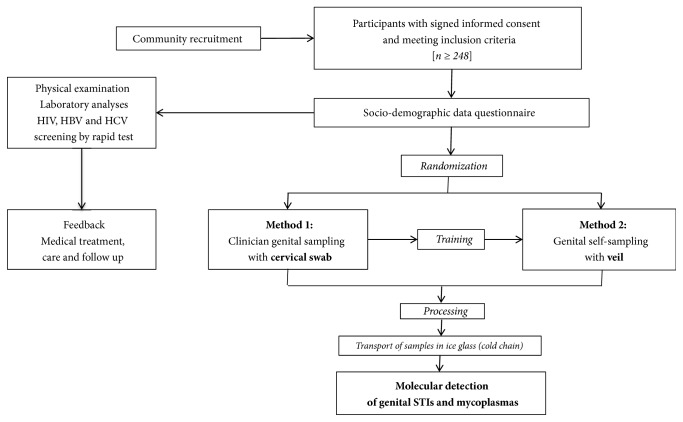
*Flow diagram of the GYNAUTO-STI study*. The* GYNAUTO-STI* study consisted in community-based recruitment of at least 248 adult women recruited from the community and further referred to the gynecologic clinic “*La Renaissance Plus*”, N'Djamena, Chad. The participants meeting the inclusion criteria were subjected to physical examination, tested for 3 chronic viral infections endemic in Chad (HIV, HBV, and HCV) by capillary-based immunochromatographic rapid test, cared when needed, and filled in the face-to-face sociodemographic questionnaire. Afterwards, the participants were trained for self-sampling collection using the V-Veil-Up Gyn Collection Device (V-Veil-Up Pharma Ltd.) and randomly submitted to the sampling procedure with Method 1 (clinician-based collection of cervical secretions by swab, as gold standard) followed by the Method 2 (self-collection by veil) or inversely. Collected samples were processed for molecular detection of genital sexually transmitted infections (STIs) and commensal* Mycoplasmas*.

Figure 1 depicts the overview of the one-time clinical visit procedures of the* GYNAUTO-STI* study.

Women eligible for the study were received by a medical staff (preferably nurse) that explained the progress of the step-by-step protocol and had them to sign the informed consent form (Figure 1).

After having signed the informed consent form, the selected women benefited from free HIV and hepatitis B (HBV) and C (HCV) testing, by multiplex HIV/HCV/HBsAg immunochromatographic rapid test (Biosynex, Strasbourg, France) [21], clinical services including gynecological examination, family planning counseling, STIs diagnosis, laboratory analysis when necessary and appropriate treatment for those suffering from gynecologic disorders, HIV, or other genital infections. All women received an information session on HIV and STIs.

At inclusion, a standardized interview was conducted at the clinic “*La Renaissance Plus*”, by experienced counselors, using a face-to-face questionnaire, to collect socio-demographic characteristics and behavioral data, including age, marital status, social occupation, education level, residence location in N'Djamena, past history of STI, HIV status, birth control method, genital hygiene during menses, sexual behavioral characteristics such as the number of lifetime sexual partners and the age at first sexual intercourse, and assessment of knowledge regarding cervical cancer and STIs.

In order to eliminate any possible bias of sampling method and timing, the participants were further randomly selected to collect the genital secretions by clinician-based swab sampling first, followed by the veil-based self-sampling after the nurse-training, or by the veil-based self-sampling first followed by the clinician-based swab sampling. Thus, after completion of the sociodemographic questionnaire, all biological specimens were sampled and processed in the following order: (i) samples specific for each patient for medical exams according to the medical prescription following the consultation; (ii) endocervical swab collected by a doctor [Method 1] or self-collection of genital secretions using the V-Veil-Up Gyn Collection Device [Method 2] according to the randomization.

The gold standard Method 1 was carried out by a doctor using a flocked swab (Copan Diagnostic Inc., California, USA). Briefly, after placing the speculum (without lubricant prior to insertion), the physician used the swab to perform cervical sampling by introducing it into the cervical canal and performing 5 rotations before being removed and immediately placed in its plastic container. The swab was then placed in the cold (ice pack).

**Figure 2:**
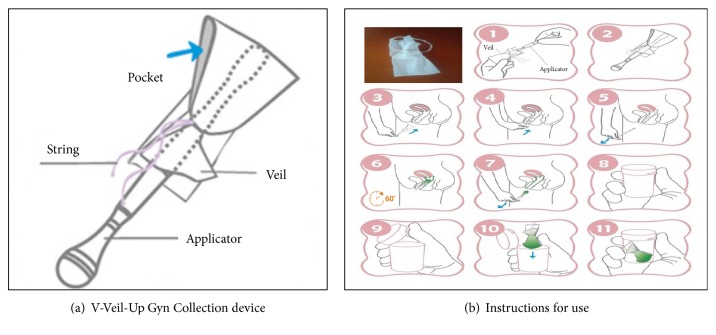
*(a) V-Veil-Up Gyn Collection Device (V-Veil-Up Pharma Ltd.)*. The V-Veil-Up Gyn Collection Device consists in a pocket (length: 75 mm, width: 45 mm, surface of contact: 6,75 cm^2^) in non-woven hydrophilic polyethylene having a capacity of absorption of 1.4 g of liquid, associated with a thread in cotton yarn (nm 16/4) and a transparent applicator (length: 120 mm, diameter: 10.8 mm) in low-density polyethylene (LD-PE). The plastic applicator makes possible to push the veil to the bottom of the vagina after its intrusion into the pocket, and the string allows the veil to be easily removed after use. The V-Veil-Up Gyn Collection Device is intended for the safe and non-invasive collection of cervicovaginal secretions from the vaginal canal. The device is conceived for self-sampling collection in privacy, and collected cervicovaginal fluid may be further gathered from the veil to be used for biological analysis. The V-Veil-Up Gyn Collection Device catches and retains safely and gently the genital secretions, thus harvesting cells, proteins, and nucleic acids (DNA/RNA) from the vaginal canal. By its conception, the device does not absorb the liquids that allowed full recovery of collected biological material, which is not trapped like in a vaginal tampon or sponge.* (b) Successive steps of the instructions for use of V-Veil-Up Gyn Collection Device*. Upon removal of the device from the vaginal canal, the sample veil is immediately protected with an opaque collection box that preserves the integrity of the genital secretions during transportation to a laboratory where downstream testing may be performed. A cap is placed on the collection box of the device to seal the unit, which is then ready for transportation to a laboratory. At least 30 minutes after taking a shower or washing the vagina, the specimens must be collected using the V-Veil-Up Gyn Collection Device. In addition, in order to avoid sampling of male genital secretions, self-sampling should be carried out after at least 2 days after unprotected sexual intercourse. In preparation for cervicovaginal collection, the contents of the device are placed on a clean and dry surface. The name or identification number of the woman and the time (date and hour) of sampling are tagged on a label sticker on the collection box. In a comfortable position, the V-Veil-Up Gyn Collection Device is taken in the dominant hand; hold the handle between the thumb and middle finger, with the index finger free above the base of the handle. With the other hand, the skin folds of the large and small lips are hold open to open the vaginal canal. The veil is then aligned with the vaginal canal by pushing the applicator. The V-Veil-Up Gyn Collection Device is then gently pressed into the vaginal canal until the contact of the cervix. It is recommended not to urinate during the time of collection. After a period of 60 minutes, the collection is considered as complete, and the veil impregnated of cervicovaginal secretions is gently removed from the vaginal canal using the string. The sample veil should then not be touched and kept clear of any other objects. Immediately after removal, the veil is placed into a 15 mL plastic box containing 10 mL of phosphate-buffered saline (PBS) solution to prevent drying of the sample, and is closed with his cap. The veil filled of secretions in its box is then placed in the cold (ice packs or crushed ice) and should be then send at best to the laboratory within 4 hours. Nota bene. The manufacturer recommends letting in place the collecting device for 30 to 60 minutes; in* GYNAUTO-STI* study, we arbitrarily chose the maximum time of sampling,* i.e.*, 60 minutes.

For the self-collection Method 2, the study participant firstly received from a nurse a 15-minutes training on how to use the V-Veil-Up Gyn Collection Device for vaginal self-sampling (Figure 2(a)). After instructing the participant, the nurse left the sampling room and the participant then performed herself the self-sampling, without any help from the nurse. The participant followed the instructions for use of the V-Veil-Up Gyn Collection Device (Figure 2(b)). Briefly, the study woman inserted the veil into her vagina, left it in-place for one hour; then removed it with the string, and returned it to the nurse. The study nurse did not witness veil insertion and removal. The nurse placed the veil impregnated with genital secretions into the dedicated collection box and closed it correctly with the cap. The veil collection box consisted of a 15 mL plastic box that contained 10 mL of phosphate-buffered saline (PBS) solution to prevent drying of the sample. The nurse verified that the PBS buffer completely submerged the veil and checked that the identification number in the label on the collection box corresponded effectively to the participant. The collection box containing the veil sample soaked with genital secretions immersed within PBS was then placed in the cold (ice pack).

### 2.4. Genital Samples Processing

Each genital sample was transported in ice packs within an hour after collection to be stored at -80°C at the virology laboratory of the* Hôpital Général de Référence Nationale*, N'Djamena, Chad. Swabs and veils were further transported in frozen ice packs to the microbiology laboratory of the* Hôpital Européen Georges Pompidou*, Paris, France, for molecular analyses.

### 2.5. Nucleic Acid Extraction

DNA was extracted from the tip of swab specimens using the DNeasyBlood and Tissue kit (Qiagen, Hilden, Germany), as recommended by the manufacturer. After extraction, DNA was concentrated in 100 *μ*L of the elution buffer provided in the extraction kit and stored at -80°C before bacterial DNA detection, as described in part previously [22].

Veil samples soaked with genital secretions within PBS buffer were carefully removed from their collection box and placed into a syringe to be drained by pulling the syringe's plunger into a 15 mL tube. The whole genital secretions were then vigorously vortexed to homogenize the fluids and finally aliquoted in 1.5 mL cryotubes (Eppendorf, Hamburg, Germany) and store at -80°C before the nucleic acid extraction procedure. In order to avoid any contamination between different specimens, the working area was sterilized between the processing of each specimen and all the consumables including gloves, syringe, forceps were for single use and were immediately discarded together with the box container. Finally, the nucleic acid extraction procedure was carried out with the DNeasy Blood and Tissue kit (Qiagen), in 1 mL of the concentrated cervicovaginal veil-collected specimen and extracted DNA was placed in 100 *μ*L of the elution buffer provided in the extraction kit and stored at -80°C before bacteria DNA detection.

### 2.6. Molecular Microbiological Analysis

Curable sexually transmitted infections [group I, including 3 bacteria (*N. gonorrhoeae*, C*. trachomatis*,* M. genitalium*) and one protozoan parasite (*T. vaginalis*)] and commensal mycoplasmas (group II, including* Mycoplasma hominis*,* Ureaplasma parvum*, and* Ureaplasma urealyticum*) were detected by molecular biology using the CE IVD-marked multiplex real-time PCR Allplex™ STI Essential Assay (Seegene, Seoul, South Korea, distributed in France by Eurobio Laboratoires, Courtaboeuf, France) [23]. The kit contains specific primers targeting each of the seven microorganism DNAs and is based on Seegene's proprietary DPO™ and MuDT™ technologies [24], which allow avoiding mismatch priming and quantifying each target in a single fluorescence channel, respectively. An internal control (*β*-globin gene) is present in the PCR mix for the detection of PCR inhibiting conditions. The multiplex PCR reaction was carried out on the CFX96™ real-time PCR instrument (Bio-Rad, Marnes-la-Coquette, France) using 5 *μ*L of extracted DNA and 15 *μ*L of PCR mix. The test was applied to all samples according to manufacturer's recommendations, using the Seegene viewer software (Seegene, South Korea) per the manufacturer's guidelines. The microbiology laboratory was accredited by the* Comité Français d'Accréditation *according to the ISO 15189 norma for molecular biology.

### 2.7. Sample Size

We hypothesized that Method 2 (veil-based self-sampling) would be non-inferior to Method 1 (clinician-based flocked swab as gold standard), with a tolerated difference of Δ in the detection rate of genital bacteria by molecular analysis between the two methods of collection. The requested minimum number (n) of subject to include was obtained by using Epi Info version 3.5.4 (CDC, Atlanta, USA), and by setting 95% confidence level, 80% statistical power, and considering estimated STIs prevalence in Chad. There exists however no data on the prevalence of genital STIs among women living in Chad. Thus, in order to estimate the genital STIs DNA positivity in our study population, we used prevalences of noncommensal genital bacterial STIs detection from comparable populations of women living in other neighboring Central African countries (Nigeria, Cameroon, Central African Republic and Sudan) previously published in the literature, including 31% for* C. trachomatis*, 3.1% for* N. gonorrhoeae* and 17.6% for* T. vaginalis *[25–31]. Based on this assumption, the estimated mean prevalence (P1) of noncommensal bacterial STIs DNA test results was 17.2% in the clinician-collected arm. The non-inferiority comparison was conducted with the hypothesis that the difference in noncommensal bacterial STIs DNA positivity between the veil-based self-collection and clinician-collection methods would be less than 10%. Finally, with Δ of 10%, the requested minimum number (n) of subject to include was at least 248 participants.

### 2.8. Statistical Analyses

Data were entered into an Excel database and analyzed using IBM® SPSS® Statistics 20 software (IBM, SPSS Inc, Armonk, New York, USA). Means and standard deviations (SD) were calculated for quantitative variables and proportions for categorical variables. The results were presented along with their 95% confidence interval (CI) using the Wilson score bounds for categorical variables. The overall prevalences of bacteria DNA detection between the two collection methods were compared using the MacNemar's test for paired data. The mean numbers of detected genital bacteria by the two collection methods were compared using the Wilcoxon signed-rank test. The agreement between the two collection methods was estimated by Cohen's *κ* coefficient, and the degree of agreement was determined as ranked by Landlis and Koch [32]. Percent agreement corresponded to the observed proportion of identical results between veil-based self-collection compared to swab-based clinician-collection. The clinician-collected microorganism DNA test results were used as the reference standard to estimate the sensitivity and specificity, with corresponding 95% CI, of the veil-collection method.

### 2.9. Ethical Approval and Consent to Participate

The Scientific Committee of the Faculty of Health Sciences of the University of N'Djamena, constituting the National Ethical Committee, formally approved the study. All included women gave their informed signed consent to participate to the study. For each included woman, the record of the consent to participate to the study was documented on each questionnaire. The Ethical Committee formally approved this consent procedure. All individual results of genital microorganism detection as well as HIV, HBV, and HCV serology were given to each study participant, and women harboring genital curable STIs were further cared for at the clinic “*La Renaissance Plus*”. Furthermore, the study results have been* in extenso* reported to health authorities of Chad during the national congress of gynecologists and midwives, held from 13 to 17 of November 2018 in the* Centre d'Etudes et de Formation pour le Développement* (CEFOD), N'Djamena, Chad.

## 3. Results

### 3.1. Characteristics of Study Population

A total of 271 women from the 23 inclusion sites accepted to participate to the study. The geographical distribution of the participants was as follow: 1^st^ district (43, 15.9%): Farcha (n=15), Amsinéné (n=13), Karkandjeri (n=15); 3^rd^ district (54, 19.9%): Gardolé (n=12), Ardep Djoumal (n=19), Kabalaye (n=10), Sabangali (n=13); 6^th^ district (47, 17.4%): Moursal (n=23), Paris-Congo (n=24); 7^th^ district (61, 22.5%): Ambata (n=5), Amtoukouin (n=6), Atrone (n=9), Boutalbagara (n=5), Chagoua (n=7), Dembé (n=8), Gassi (n=9), Habena (n=9), Kilwiti (n=3); and the 9^th^ district (66, 24.3%): Digangali (n=10), Gardolé (n=7), Ngueli (n=11), Toukra (n=12), and Walia (n=26).

**Figure 3:**
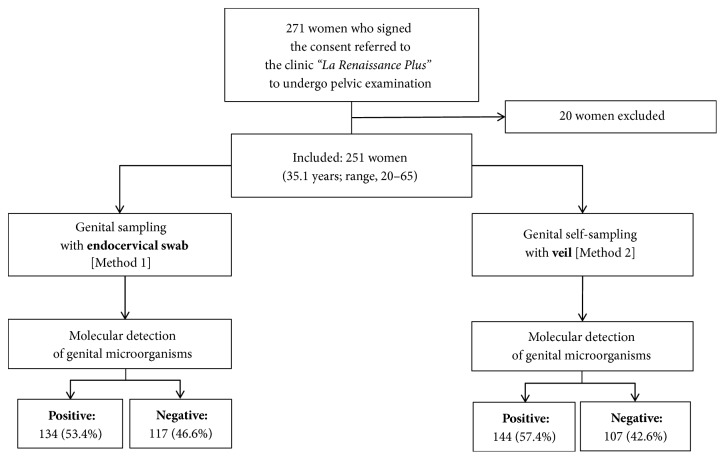
Flow diagram of study recruitment, specimen collection, and results of molecular detection by multiplex real-time PCR of genital microorganisms, including common curable sexually transmitted infections (STIs) and commensal mycoplasmas.

After physical examination, 20 women were excluded because of genital troubles (vaginal discharge: 8; genital bleeding: 6; sexual intercourse less than 2 days: 6). Finally, a total of 251 clinically asymptomatic women (mean age, 35.1 years; range, 18–65) referred to the clinic “La Renaissance Plus” were consecutively and prospectively included in the study (Figure 3). Their sociodemographic characteristics, past history of STIs, sexual behavior, contraception, and practices of feminine hygiene during menstruation and genital toilet are summarized in the Table 1.

Using multiplex HIV/HCV/HBsAg rapid test, 9 study women (3.6%) were infected by HIV-1 and 19 (7.6%) by HBV (positivity for HBsAg) and 8 (3.2%) were seropositive for HCV.

Most women (31.1%) were young, aged from 18 to 29 years, engaged in life coupled with a male partner (80.9%), with a relatively high education level (64.2%); most of them were unemployed (54.6%). The majority of study women (80.1%) reported having only one regular sexual partner in their life, while 16.3% reported to have had up to 5 different sexual partners. Generally, the study participants began sexual activity before 17 years (54.2%). The vast majority of women (73.7%) did not take any birth control methods. Concerning the feminine hygiene during menstruation, most women (92.8%) were using sanitary napkins, while a minority (7.2%) used commercially available tampons. Genital (vulva or vagina) toilet was the rule, including post-coital toilet with water and finger in 91.6%.

### 3.2. Curable STIs and Genital Mycoplasmas Detection and Distribution by Collection Methods

**Figure 4:**
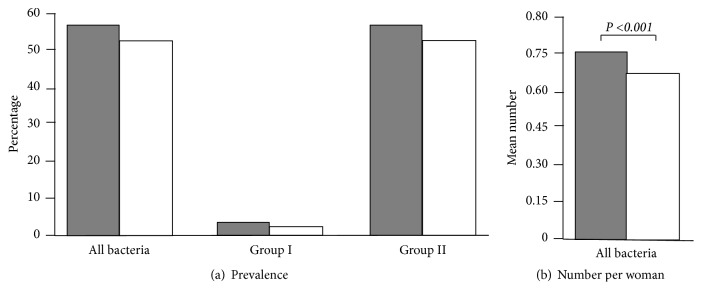
Percentages of detection (a) and mean number of genital microorganisms detection (b) by multiplex real-time PCR Allplex™ STI Essential Assay (Seegene) of genital bacteria, including common curable sexually transmitted infections (group I) and commensal mycoplasmas (group II) in paired genital secretions obtained by gold standard clinician-collected endocervical swab (white) and by self-collection using the V-Veil-Up Gyn Collection Device (V-Veil-Up Pharma Ltd.) (grey) among 251 study women living in N'Djamena, Chad.* P*-values of the comparison between the percentages of detection by the two collection methods using the Mac Nemar's test for paired data were not significant for all comparisons.* P*-value of the comparison between the mean numbers of detected genital bacteria by the two collection methods using the Wilcoxon signed-rank test is indicated in italic.

All 251 study participants had paired clinician-collected and self-collected specimens obtained for laboratory testing. All secretions from swab and veil specimens were positive for the *β*-globin gene. Results from each of the collection method are depicted in Tables 2 and 3 and in Figures 3 and 4.

Of the clinician-collected specimens included in the analysis, 134 women were positive for genital microorganisms giving a total prevalence of 53.4% (95% CI: 47.2–59.6) (Table 2; Figures 3 and 4). Six (2.4%, 95% CI: 0.5-4.3) women were infected with at least one common STI.* C. trachomatis*,* N. gonorrhoeae*,* M. genitalium*, and* T. vaginalis* were recovered from 2 (0.8%), 1 (0.4%), 4 (1.6%), and 1 (0.4%) participants, respectively, and a combination of two pathogens was found in two women (*M. genitalium* plus* N. gonorrhoeae*: 1 (0.4%), 95% CI: 0.0-1.2; and* M. genitalium* plus* C. trachomatis*: 1 (0.4%), 95% CI: 0.0-1.2). A total of 134 (53.4%, 95% CI: 47.2-59.6) women carried at least one genital mycoplasma, and* U. parvum* was the most represented species and was present alone or associated with other species in 42.6% of participants. Combinations of two or three species were observed in 21 (8.4%) and 4 (1.6%) women, respectively.* M. hominis* associated with* U. parvum* was the most frequent association.

Of the veil-based self-collected specimens included in the analysis, 144 women were positive for genital microorganisms giving a total prevalence of 57.4% (95% CI: 51.2–63.5) (Table 2; Figures 3 and 4). Seven (2.8%, 95% CI: 0.7-4.8) women were infected with at least one common STI.* C. trachomatis*,* N. gonorrhoeae*,* M. genitalium*, and* T. vaginalis* were recovered from 3 (1.2%), 1 (0.4%), 4 (1.6%), and 1 (0.4%) participants, respectively, and a combination of two pathogens was found in two women (*M. genitalium* plus* N. gonorrhoeae*: 1 (0.4%), 95% CI: 0.0-1.2; and* M. genitalium* plus* C. trachomatis*: 1 (0.4%), 95% CI: 0.0-1.2). A total of 144 (57.4%, 95% CI: 51.2-63.5) women carried at least one genital mycoplasma, and* U. parvum* was the most represented species, alone or associated with other species in 44.2% of participants. Combinations of two or three species were observed in 30 (11.9%) and 3 (1.2%) women, respectively.* M. hominis* associated with* U. parvum* was the most frequent association. One woman (#009) showed 4 genital bacteria (*C. trachomatis*,* M. genitalium*,* M. hominis*, and* U. parvum*) by veil (Table 4).

The results of the comparison of the two collection methods are detailed in the Table 3. The percent agreements between the two collection methods to detect any genital microorganisms, common curable sexually transmitted infections (group I) and commensal mycoplasmas (group II) were 92.8%, 99.6%, and 92.8%, respectively, and all Cohen's *κ* coefficients were between 0.81 to 0.99, demonstrating “almost perfect” agreement [32].

In addition, using clinician-collected swab as the reference collection method, the sensitivities and specificities of the self-collected veil to detect any genital microorganisms and commensal mycoplasmas (group II) were 97.0% and 88.0% (Table 3).

Overall, the percentages of test positivity for any genital microorganism, common curable sexually transmitted infections (group I) and commensal mycoplasmas (group II) showed a trend to be slightly higher in self-collected specimens than clinician-collected specimens, but the trends were not statistically significant for all comparisons (57.4%* versus* 53.4%;* P*=0.9; 2.8%* versus* 2.4%;* P*=1.0; and 57.4%* versus* 53.4%;* P*=0.9) (Figure 4(a)). Interestingly, the mean total number of genital microorganisms detected per study woman by veil-based self-collected method was 1.14-fold slightly higher than by clinician-collected method [0.75±0.77 (188/251; range, 0-4)* versus* 0.66±0.71 (165/251; range, 0-3);* P*<0.001] (Figure 4(b)).

The Table 4 depicts some relevant examples of paired results obtained by clinician-collected swab and self-collected veil using the multiplex real-time PCR Allplex™ STI Essential Assay.

## 4. Discussion

The present study reports on the prevalences of common curable STIs (*N. gonorrhoeae*,* C. trachomatis*,* M. genitalium*, and* T. vaginalis*) as well as genital mycoplasmas (*M. hominis*,* U. urealyticum*, and* U. parvum*) colonization among community-recruited asymptomatic adult women living in N'Djamena, Chad. The results showed low incidence of common STIs, as only seven women (2.8%) were infected. The prevalences of genital mycoplasmas (apart from* M. genitalium*) were much higher in more than half women with a predominance of* U. parvum*. Furthermore, the genital microorganisms DNA diagnostic accuracy of a novel genital veil (V-Veil-Up Gyn Collection Device) was assessed as female genital self-sampling device to collect cervicovaginal secretions. All specimens collected by the veil were found positive for the *β*-globin gene, demonstrating the lack of PCR inhibitors or the presence of cellular DNA, which made STIs detection possible by molecular testing. The results showed high accuracy of the veil-based genital self-collection, which was non-inferior to clinician-based collection as reference for genital bacteria DNA molecular testing, with “almost perfect” agreement between the two collection methods, high sensitivity and specificity. Outstandingly, the mean number of detected genital bacteria was significantly higher when using veil-based collected genital secretions than clinician-collected cervical secretions by swab. Thus, the self-collection by veil allowed detecting 1.14-fold more genital bacteria than the swab-based collection, likely originating from cervical as well as non-cervical areas of the vaginal cavity, including vaginal cul-de-sacs, vaginal walls, and vulva. Taken together, veil-based self-collection of genital secretions appears a convenient tool to collect in a gentle way genital secretions for accurate molecular detection of female genital bacteria. Such sampling procedure could be easily implemented in STIs clinics in sub-Saharan Africa.

Epidemiological data on the prevalences of common bacterial STIs in Chad have been until now poorly reported. In a cohort of 311 HIV-1-seropositive women, Mortier and colleagues found a prevalence of 1% for* T. vaginalis* [33]. Remarkably, the prevalences of genital shedding of* C. trachomatis*,* N. gonorrhoeae*,* M. genitalium*, and* T. vaginalis *were particularly low in study women. In neighboring countries, such as Nigeria, Cameroon, Central African Republic, and Sudan, various prevalence rates of female genital recovery of* C. trachomatis* (0.7 – 31%),* N. gonorrhoeae* (0–3.1%), and* T. vaginalis* (0.4 – 17.6%) have been reported [25–31]. This high heterogeneity among prevalences of common STIs in adult women populations living in Central Africa could be likely explained by the wide variations in study settings and populations and laboratory methods used for genital sampling and STIs screening [34].

The high rate of carriage of genital mycoplasmas in our study population is reminiscent of previous reports in women of reproductive age from neighboring countries, with prevalence rates of 65% in Cameroon [35] and 36% in Nigeria [36]. Genital mycoplasmas apart from* M. genitalium* (including* M. hominis*,* U. parvum*, and* U. urealyticum*) are generally considered as commensals of the female uro-genital tract, as they may be found at rates up to 80% in sexually active women [37, 38]. However, the high prevalence of genital mycoplasmas apart from* M. genitalium *in this population would need further investigation to assess its clinical consequences and potential adverse pregnancy outcomes.

Taking into account that most adult Chadian women are living in remote rural areas, or far away of adequate healthcare facilities, self-collection of genital specimen carried out at home by women themselves could represent a relevant alternative allowing increasing the coverage of screening when coupled with adapted STI pathogens testing by molecular biology. In the* GYNAUTO-STI* study, we have had the opportunity to evaluate the genital microorganisms DNA diagnostic accuracy of a novel genital veil (V-Veil-Up Gyn Collection Device) as female genital self-sampling device to collect cervicovaginal secretions.

The percent of agreements between clinician-based collection and veil-based self-collection to detect any vaginal microorganisms was above 90%, and all Cohen's *κ* coefficients were between 0.81 to 0.99 demonstrating “almost perfect” agreement between collection methods [32]. Although no data exists on the performance of self-collected specimens by veil for female genital bacteria DNA testing for which to compare our results, several previous reports evaluating the use of self-collection by vaginal swab for* C. trachomatis* and* N. gonorrhoeae* testing may be useful to contextualize our observations [14]. A recent meta-analysis showed a lower proportion of positive* C. trachomatis* or* N. gonorrhoeae* test results with home-based collection by vaginal swab (240/2074, 11.6%) than with clinic-based (179/967, 18.5%) specimen collection (risk ratio of 0.72, 95% CI 0.61 to 0.86; number of participants: 3041) [8, 14, 16, 39–44]. In contrast to self-sampling by vaginal swab, our results demonstrate that self-sampling by veil allows detecting by molecular biology similar rates of common curable STIs and commensal mycoplasmas as clinician-based collection by cervical swab. These observations are in keeping with the report by Cook and colleagues who reported equivalent rates of positive tests for* C. trachomatis* or* N. gonorrhoeae* per 100 woman-years of follow-up for home-collected and clinic-collected specimens (20.4* versus* 24.1;* P*=0.28) in a prospective randomized controlled trial including high-risk young women followed up during 18 months [12]. Finally, the “almost perfect” agreement between both collection methods was corroborated by elevated sensitivities of self-collected veil to detect by molecular biology any genital microorganisms and commensal mycoplasmas.

Because clinical management and research usually depend on single-point detection, it is important to use the collection method the most capable to detect genital microorganisms by molecular biology. For example, the cumulative presence of HPV in female genital tract is always greater than its point prevalence, suggesting that single-point sampling is less than 100% sensitive [45]. Otherwise, the vaginal epithelium represents a much greater surface area than the cervical epithelium. Thus, the self-sampling approach using veil might not only sample the cervix, but it will also sample the vaginal epithelium, with a potentially greater likelihood of detecting genital microorganisms. In the present study, the percentages of test positivity for genital microorganisms showed a trend to be slightly higher in self-collected than clinician-collected specimens, however without statistical significance. Remarkably, in the present study, the mean total number of genital microorganisms detected per study woman was 1.14-fold higher in self-collected specimens by veil than in clinician-collected specimens. These findings suggest that the veil is able to significantly release higher amount of genital secretions for molecular analysis than cervical swab. In fact, female genital secretions embedded in a rayon-covered cotton core tampon are not easily separated from the sampling device [46]. Our results show the high capacity of the veil to allow the detection of genital microorganisms present within the vaginal cavity that could not be detected from the paired swab specimens. Indeed, the veil collects all types of cervicovaginal secretions of the vaginal cavity, not exclusively the cervical secretions, and thus allows also detecting microorganisms* a priori* located at non-cervical vaginal areas (vaginal cul-de-sacs, vaginal walls and vulva).

Our observations also point to the potential interest of using a supervised self-collection strategy among African women, in which oral counseling processes are aided at all times by a healthcare or non-healthcare professional as a counselor to understand the instructions for use, the genital anatomy, and provide counseling with a very high rate of acceptability and satisfaction of self-collection. The evidence of high acceptability for supervised strategies was previously reported for another self-collection of capillary blood or saliva during HIV self-testing, especially in resource-constrained settings [47]. Finally, providing options for self-collection based upon women's preferences is likely to increase screening coverage. However, whether face to face instructions may become a practical barrier for future routine use of veil-based self-sampling should be the subject of a thorough field evaluation in African context.

Our study had several strengths. First, we tried to limit the selection bias of the study population by community-based recruitment in order to make this survey as much representative as possible of the female population in Chad. Thus, study participants referred to clinic “*La Renaissance Plu*s”, N'Djamena, were not patients from the clinic, avoiding the obvious bias of recruitment by health care facilities. Furthermore, 9 (3.5%), 19 (7.5%), and 8 (3.2%) study participants were HIV-, HBsAg-, and HCV-specific antibody positive, respectively, in accordance with the high endemicity of these three major chronic viral infections in Chad [48, 49]. However, our study has limitations. First, although 251 women were enrolled to the study, the insufficient size of the study population may have introduced bias. The fact that women participated on a voluntary basis and were recruited from community-churches and mosques and associative networks may also have led to recruitment bias. Secondly, we had to rely on a face-to-face questionnaire to collect sociodemographic data and information on sexual behaviors, a method that may be prone to reporting bias for sensitive and intimate questions [50]. Thirdly, we did not evaluate the acceptability of the veil in this study, especially in the African cultural context. However, it is established that self-sampling to diagnose curable STIs in adult women is feasible and acceptable with the majority having a positive experience and willingness to use again [7, 13], even in low-income communities like in sub-Saharan Africa [16, 51, 52]. Finally, the performance of the genital veil as self-sampling device to diagnose STIs by PCR was carried out in a group of adult women clinically asymptomatic for STIs that allows certainly accurate assessment of its specificity but only a partial assessment of its sensitivity.

In conclusion, the diagnosis and prevention of bacterial STIs is likely one of the most important public health challenges that Chad has to face in a near future. Self-collection of genital secretions using the V-Veil-Up Gyn Collection Device constitutes a simple and powerful tool to collect genital secretions for further molecular testing and screening of genital microorganisms that could be easily implemented in the national prevention program in Chad. In regions of the world where access to care is limited due to socioeconomic reasons or where clinician-collected samples may be limited due to personal and/or sociocultural concerns, self-collection method by inexpensive veil may provide a way to extend screening to an underserved population.

## Figures and Tables

**Table 1:** Sociodemographics, behavioural, and hygiene characteristics of the 251 women living in N'Djamena, Chad, and recruited from the community.

Variable/category	All study women (N = 251)
	*n (*%*) [95% CI]*^*∗*^
*Age* (years)	
	78 (31.1) [25.4–37.2]
≥ 30 − < 40	66 (26.3) [21.0–32.2]
≥ 40 − < 50	69 (27.5) [22.1–33.5]
≥ 50	38 (15.1) [10.9–20.2]
*Marital status*	
Married or living in couple	203 (80.9) [75.5–85.6]
Single	48 (19.1) [14.4–24.5]
Occupation	
Unemployed	137 (54.6) [48.2–60.9]
Student	36 (14.3) [10.3–19.3]
Employed	78 (31.1) [25.4–37.2]
*Education level*	
Never schooled	47 (18.7) [14.1–24.1]
Elementary school	43 (17.1) [12.7–22.4]
High school	80 (31.9) [26.2–38.0]
University	81 (32.3) [26.5–38.4]
*Age at first sexual intercourse (year)*	
≤ 17	136 (54.2) [47.8–60.5]
≥ 18	110 (43.8) [37.6–50.2]
No response	5 (2.0) [0.6–4.6]
*Number of sexual partners in the life*	
One regular partner	201 (80.1) [74.6–84.8]
Several partners [1 to 5]	41 (16.3) [12.0–21.5]
*Past history of STIs* ^*∗∗*^	
Yes	11 (4.4) [2.2–7.7]
No	240 (95.6) [92.3–97.8]
*HIV-1 status*	
Positive	9 (3.6) [1.7–6.7]
Negative	242 (96.4) [93.3–98.3]
*HBV status*	
Positive	19 (7.6) [4.6–11.6]
Negative	232 (92.4) [88.4–95.4]
*HCV status*	
Positive	8 (3.2) [1.4–6.2]
Negative	243 (96.8) [93.8–98.6]
*Vaginal tampon use*	
Never^*∗∗∗*^	215 (85.7) [80.7–89.7]
Sometimes^*∗∗∗*^	14 (5.6) [3.1–9.2]
Often^*∗∗∗*^	4 (1.6) [0.4–4.0]
Always	18 (7.2) [4.3–11.1]
*Regular genital toilet*	
Yes	201 (80.1) [74.6–84.8]
Water	110 (54.7) [47.6–61.7]
Water + soap	91 (45.3) [38.3–52.4]
No	50 (19.9) [15.2–25.4]
*Postcoital genital toilet*	
Yes	230 (91.6) [87.5–94.7]
Water	141 (61.3) [54.7–67.6]
Water + soap	89 (38.7) [32.4–45.3]
No	21 (8.4) [5.3–12.5]
*Use of contraceptive*	
Yes	66 (26.3) [21.0–32.2]
Pill	17 (25.8) [15.8–38.0]
Intrauterine device	11 (16.7) [8.6–27.9]
Condom	7 (10.6) [4.4–20.6]
Other	31 (47.0) [34.6–59.7]
No	185 (73.7) [67.8–79.0]

^*∗*^The frequency of each variable is presented with their 95% confidence interval in brackets.

^*∗∗*^Including infections due to *N. gonorrhoeae*, *C. trachomatis*, and syphilis.

^*∗∗∗*^Alternative of vaginal tampon use for feminine hygiene during menstruation was the use of sanitary napkins.

CI: confidence interval; HIV-1: human immunodeficiency virus; HBV: hepatitis B virus; HCV: hepatitis C virus; STIs: sexually transmitted infections.

**Table 2:** Prevalences of common curable sexually transmitted infections (group I) and commensal mycoplasmas (apart from *M. genitalium*) (group II) in the genital tract of 251 adult women living N'Djamena, Chad, and recruited from the community.

	All study women (N=251) whatever the collection methods *n (%)*	Veil-based self-collection *n (%) [95% CI]*^*∗*^	Swab-based clinician-collection *n (%) [95% CI]*^*∗*^	*P* ^*∗∗*^
*Group I: Curable sexually transmitted bacteria*				
*Chlamydia trachomatis*	3 (1.2)	3 (1.2) [0.0-2.5]	2 (0.8) [0.0-1.9]	1.0
*Neisseria gonorrhoeae*	1 (0.4)	1 (0.4) [0.0-1.2]	1 (0.4) [0.0-1.2]	1.0
*Mycoplasma genitalium*	4 (1.6)	4 (1.6) [0.04-3.1]	4 (1.6) [0.04-3.1]	1.0
*Trichomonas vaginalis*	1 (0.4)	1 (0.4) [0.0-1.2]	1 (0.4) [0.0-1.2]	1.0
At least 1 curable STI	7 (2.8)	7 (2.8) [0.0-1.9]	6 (2.4) [0.5-4.3]	1.0
At least 2 curable STIs	2 (0.8)	2 (0.8) [0.0-1.9]	2 (0.8) [0.0-1.9]	1.0

*Group II: Commensal mycoplasmas *				
*Mycoplasma hominis*	34 (13.5)	30 (11.9) [7.9-15.9]	26 (10.4) [6.6-14.1]	0.7
*Ureaplasma urealyticum*	37 (14.7)	36 (14.3) [10.1-18.7]	28 (11.2) [7.2-15.1]	0.3
*Ureaplasma parvum*	115 (45.8)	111 (44.2) [38.1-50.4]	107 (42.6) [36.5-48.7]	0.8
At least 1 commensal mycoplasma	150 (59.7)	144 (57.4) [51.2-63.5]	134 (53.4) [47.2-59.6]	0.9
At least 2 commensal mycoplasmas	34 (13.5)	30 (11.9) [7.3-15.1]	21 (8.4) [5.6-12.7]	0.9

^*∗*^The frequency of each variable is presented with their 95% confidence interval in brackets.

^*∗∗*^Statistical comparisons were assessed by McNemar test for paired data.

CI: confidence interval; STI: sexually transmitted infection.

**Table 3:** Two-by-two tables of cervicovaginal specimens self-collected using the V-Veil-Up Gyn Collection Device (V-Veil-Up Pharma Ltd.) compared to clinician-collected endocervical swab specimens for the detection by multiplex real-time PCR of genital bacteria, including common curable sexually transmitted infections (group I) and commensal mycoplasmas (group II) among 251 adult women living in N'Djamena, Chad, and recruited from the community.

		*All genital bacteria*			*Group I*			*Group II*	

		*Clinician-collected swab specimen*			*Clinician-collected swab specimen*			*Clinician-collected swab specimen*	

		*Positive* (N=134)	*Negative* (N=117)		*Positive* (N=6)	*Negative* (N=245)		*Positive* (N=134)	*Negative* (N=117)

*Veil- based* *self-collected specimen*	*Positive* (N^*∗*^=144)	130	14	*Positive* (N^*∗∗∗*^=7)	6	1	*Positive* (N^$^=144)	130	14
	*Negative* (N^*∗∗*^=107)	4	103	*Negative* (N^*∗∗∗∗*^=244)	0	244	*Negative* (N^£^=107)	4	103

		*Estimate*	*95*%* CI*		*Estimate*	*95*%* CI*		*Estimate*	*95*%* CI*

*Sensitivity (*%)		97.0	[92.5-99.2]		NA^+^	-		97.0	[92.5-99.2]

*Specificity (*%)		88.0	[80.7-93.3]		NA^+^	-		88.0	[80.7-93.3]

*Agreement (*%)		92.8			99.6			92.8	

*Cohen's κ coefficient* ^*μ*^		0.85			0.92			0.85	

^*∗*^Total number of veil based-self collected specimens positive for all genital microorganisms.

^*∗∗*^Total number of veil based-self collected specimens negative for all genital microorganisms.

^*∗∗∗*^Total number of veil based-self collected specimens positive for genital microorganisms of Group I.

^*∗∗∗∗*^Total number of veil based-self collected specimens negative for genital microorganisms of Group I.

^$^Total number of veil based-self collected specimens positive for genital microorganisms of Group II.

^£^Total number of veil based-self collected specimens negative for genital microorganisms of Group II.

^*μ*^The Cohen's *κ* coefficient was interpreted according the Landis and Koch scale [32]: For *κ* value 0, the agreement is considered to be less than what would be expected by chance; for *κ* values 0.01–0.20, only a “slight agreement” is present; for *κ* values 0.21–0.40, the agreement is considered to be “fair”; for *κ* values 0.41–0.60, the agreement is said to be “moderate”; for *κ* values 0.61–0.80, the agreement is considered “good”; and finally, for *κ* values 0.81–0.99, the agreement is said to be “almost perfect”.

^+^NA: not attributable (because of the low common STIs positivity rate (<3%) in study population which makes impossible to calculate an accurate estimate of sensitivity and specificity for the group I).

**Table 4:** Relevant selected examples of paired results obtained by gold standard clinician-collected endocervical swab and self-collected veil using the multiplex real-time PCR Allplex™ STI Essential Assay (Seegene). Participants #021, #036, and #233 gave similar profiles of bacteria detection by paired clinician-collected swab and self-collected veil. Participants #058 and #152 showed more detection by swab than by veil. Finally, participants #009, #041, #090, #126, #172, #173, #177, and #231 showed better bacteria detection by self-collected veil than clinician-collected swab. Positive result for a given bacteria is indicated by cross whereas negative result by white box. All swab and veil specimens were positive for *β*-globin internal control of the PCR Allplex™ STI Essential Assay (not shown). Group I corresponds to common curable sexually transmitted infections (*Chlamydia trachomatis*, *Neisseria gonorrhoeae*, *Mycoplasma genitalium*, and *Trichomonas vaginalis*) and group II to commensal mycoplasmas (*Mycoplasma hominis*, *Ureaplasma urealyticum, *and* Ureaplasma parvum*).

Specimens	Genital microorganisms detection						
	Group I				Group II		
	*C. trachomatis*	*N. gonorrhoeae*	*M. genitalium*	*T. vaginalis*	*M. hominis*	*U. urealiticum*	*U. parvum*
#009							
Veil	+		+		+		+
Swab	+		+				+

#021							
Veil				+			+
Swab				+			+

#036							
Veil		+	+			+	
Swab		+	+			+	

#041							
Veil					+		
Swab							

#058							
Veil			+				
Swab			+		+		+

#090							
Veil					+	+	
Swab							

#126							
Veil						+	+
Swab							+

#152							
Veil							
Swab					+		+

#172							
Veil							+
Swab							

#173							
Veil					+	+	+
Swab					+		+

#177							
Veil	+						+
Swab							+

#231							
Veil					+	+	
Swab							

#233							
Veil	+				+		+
Swab	+				+		+

## Data Availability

The datasets analyzed during the current study are available from the corresponding author upon reasonable request.

## Abbreviations

COFRAC: Comité Français d'Accréditation
CEFOD: Centre d'Etudes et de Formation pour le Développement
HBV: Hepatitis B virus
HCV: Hepatitis C virus
HIV: Human immunodeficiency virus
HPV: Human papillomavirus
STI: Sexually transmitted infection
WHO: World Health Organization.
